# Supplementary material for: Meta-analysis of nationwide SARS-CoV-2 infection fatality rates in India
Source: PLOS Glob Public Health. 2022 Sep 19;2(9):e0000897. doi: 10.1371/journal.pgph.0000897 (PMC10021252; doi:10.1371/journal.pgph.0000897)
Supplement: S1 Text — (DOCX) [file pgph.0000897.s002.docx]

**Supplementary Online Content**

**Meta-analysis of nationwide SARS-CoV-2 infection fatality rates**

**in India**

**Methods A in S1 Text.** Systematic review search strategy

**Methods B in S1 Text.** Meta-analysis methodology

**Methods C in S1 Text.** Meta-analysis framework

**Methods D in S1 Text.** Risk of bias assessment among included articles

**Methods E in S1 Text.** Assessment of publication bias

**Table A in S1 Text.** PRISMA checklist

**Table B in S1 Text.** Summary of regional mortality data from subnational studies in India from 2020-2021

**Fig A in S1 Text.** Description of data extraction

**Fig B in S1 Text.** Funnel plot for assessment of publication bias among included studies

**References**

This supplemental material has been provided by the authors to provide readers with additional details about their research.

**Methods A in S1 Text.** Systematic review search strategy

The original search was conducted on July 3, 2021, reflecting published studies and preprints available from January 1, 2020 to July 3, 2021, which also contains the published full search concept blocks and filters applied [1]*.* A second search was performed through August 15, 2021 [1] and a third search was performed through October 15, 2021. A fourth updated search verification presented herein encompasses published articles and preprints made available from October 1, 2021 to May 26, 2022 in the following ten journals/databases: medRxiv, *BMJ Global Health*, *Indian Journal of Medical Research*, *International Journal of Epidemiology*, *International Journal of Infectious Diseases*, *JAMA*, *Lancet*, *PloS*, *Nature*, and *Science*.

**Methods B in S1 Text.** Meta-analysis methodology

*(Note: Details below are similar to the Supplementary Materials in previous submission* [1] *with some exceptions, as noted.)*

The aim of the meta-analysis is to estimate a nationwide IFR_2_ for SARS-CoV-2 in India that captures both underreporting of cases, based on seroprevalence studies, and underreporting of deaths, based on countrywide excess deaths studies and disease transmission-based studies.

***Data collection and preparation***

For included studies with a pre-calculated infection fatality rate that is as characterized above, the IFR_2_, along with the 95% confidence interval, are directly extracted from the included study.

For studies without a pre-calculated IFR_2_, IFR_2_ is computed, as given in the below formula

$$IFR_{2}=\frac{\boldsymbol{Estimated} Total Cumulative Deaths}{\boldsymbol{Estimated}Total Cumulative Infections} (a)$$

where $\boldsymbol{Estimated}Total Cumulative Infections=Seroprevalence*Age Adjusted Population$, $\boldsymbol{Estimated} Total Cumulative Deaths=All-Cause Mortality Excess Deaths$ for excess death studies and $\boldsymbol{Estimated} Total Cumulative Deaths=COVID-19 Related Deaths$ for disease transmission-based studies.

For the denominator in formula $(a)$, the seroprevalence estimate corresponding to the general study population, as well as the 95% confidence interval, are directly extracted from the 4^th^ nationwide serosurvey for India [2]. We retrieved seroprevalence estimates that were adjusted (within the serosurvey design) for the test performance and weighted to be representative of the study’s general population (typically, for the demographics age and sex, among others, such as rural versus urban), if available. The age-adjusted population estimate is calculated as the 2019 projected population estimate on the 2011 census website multiplied by the proportion of the population above the age-cutoff of the included study (e.g., proportion of the population of India aged ≥ 6 years), as obtained from the age composition for the study area from the 2011 census. For the numerator in formula $(a)$, the deaths estimate is either directly extracted from excess deaths studies or disease transmission-based studies through at least June 2021 and extending to December 2021, available at the time of this report. When an included study provided multiple death estimates that are derived from distinct/independent data sources and are through at least June 2021, we include all available death estimates as data points in the meta-analysis. If multiple versions of the same study are returned by the search, we consider the latest version of the study in the qualitative and quantitative synthesis, respectively.

Being that the infection fatality rate (IFR) measure is understood to be a rate and that upon inspection its distribution was heavily right skewed, a log transformation is applied to the sampling data to achieve a unimodal distribution with more evenly distributed tails.

For the 95% confidence interval (CI) for ${IFR}_{2}$, first we obtain the standard error for seroprevalence from the upper and lower CI bounds of the directly provided 95% CI from each included study as

$se_{sero}=\frac{Upper CI-Lower CI}{1.96*2}=\frac{Upper CI-Lower CI}{3.92} (b)$

Next, when the included study provides a 95% confidence interval for the excess deaths estimates, we obtain the standard error for the excess deaths estimates in a similar manner from the directly provided 95% CI from each included study as

$se_{log deaths}=\frac{log\{Upper CI\}-log\{Lower CI\}}{1.96*2}=\frac{log\{Upper CI\}-log\{Lower CI\}}{3.92} (c)$

Alternatively, when a confidence interval for the excess deaths estimates is not provided, we obtain the 95% confidence interval generically by adding and subtracting two standard deviations from an assumed standard normal distribution, where the Z statistic for 95% CI is z = 1.96 (i.e., the 97.5 percentile value from the standard normal distribution).

Now that we have the standard error for seroprevalence and excess deaths, the standard error for the log of IFR_2_ can be obtained as IFR_2_ relies on $Seroprevalence$ and $Total Cumulative Deaths$ as detailed in formula $(a)$ above. First, notice that

$$\log\left( {IFR}_{2} \right)=\log\left\{ \frac{Total Cumulative Deaths}{Total Cumulative Infections} \right\}$$

By rules of logarithmic operations, it follows that

$$=\log\left( Total Cumulative Deaths \right)-\log\left( Total Cumulative Infections \right)$$

By the definition in formula $(a)$, it follows that

$$=\log\left( \hat{d} \right)-\log\left( n*\hat{p}_{sero} \right)$$

where $\hat{d}$ is the estimated deaths from the included study, $n$ is the study sample size from the serosurvey and $\hat{p}_{sero}$ is the seroprevalence estimate from the serosurvey.

By rules of logarithmic operations, we have that

$$=\log\left( \hat{d} \right)-\log\left( n)-log(\hat{p}_{sero} \right)$$

Assuming $n$ does not contribute to variability and fixing at some constant, it follows that

$$=C+ log \left( \hat{d} \right)-\log\left( \hat{p}_{sero} \right)$$

Therefore, $\log\left( {IFR}_{2} \right)= C+ \log\left( \hat{d} \right)- \log\left( \hat{p}_{sero} \right).$Let us consider the variance of $\log\left( {IFR}_{2} \right).$

Substituting in for $\log\left( {IFR}_{2} \right)$ from above, we have

$$Var(log {IFR}_{2})= Var\left( log \hat{d}-log \hat{p}_{sero} \right)$$

Since the studies are assumed to be independent, the purported covariance term is zero and we have that

$$Var\left( log \hat{d}-log \hat{p}_{sero} \right)=Var\left( log \hat{d} \right)+Var\left( log \hat{p}_{sero} \right)$$

From the Taylor Series expansion, it follows that

$$Var\left( \log\hat{p}_{sero} \right) \approx\frac{1}{\hat{p}_{sero}^{2}} Var\left( \hat{p}_{sero} \right)$$

Then, the standard error for the log of IFR_2_ is given by

$se_{log IFR_{2}}\approx$ $\sqrt{\left\{ Var\left( log \hat{d} \right)+Var\left( log \hat{p}_{sero} \right) \right\}}$

$\approx$ $\sqrt{\left\{ se_{log deaths}^{2}+\frac{1}{\hat{p}_{sero}^{2}} Var\left( \hat{p}_{sero} \right) \right\}}$

where $se_{sero}$ and $se_{log deaths}$ are defined as in formulas $(b)$ and $(c)$above, respectively. Additionally, $\hat{p}_{sero}$ is the directly provided seroprevalence estimate.

Then, letting $\hat{\theta}$ denote the estimate of$\log{IFR}_{2}$, the asymptotic approximate 95% confidence interval for $\log IFR_{2}$is as follows:

($\hat{\theta}-1.96*se_{log IFR_{2}}$,$\hat{\theta}+1.96*se_{log IFR_{2}}$)

The resulting confidence intervals are then exponentiated to back-transform from the logarithmic scale.

**Methods C in S1 Text.** Meta-analysis framework

A random effects model is used with the DerSimonian-Laird (DL) estimator for $\tau^{2}$ (also denoted as $tau^{2}$), the variance of the true effect sizes. The DL estimator, $\hat{\tau_{DL}^{2}}$, is given by

$$\hat{\tau_{DL}^{2}}=\max\left\{ 0,\frac{Q_{w}-\left( k-1 \right)}{\left[ \sum_{i} w_{i}-\left( \frac{{\sum_{i} w_{i}}^{2}}{\sum_{i} w_{i}} \right) \right]} \right\} (d)$$

where $Q_{w}$ denotes the appropriate test statistic with $k$*-1* denoting the degrees of freedom and $w_{i}$ denotes the sampling weight for the $i^{th}$ included study datapoint.

The inverse variance approach is then used to obtain the pooled estimate that is nationwide for India. This means that the weighting in the random effects model is the inverse of the sampling variance, as follows

$$w_{i}^{\left\{ DL \right\}}=\frac{1}{se_{i}^{2}+\hat{\tau_{DL}^{2}}} (e)$$

where $i$ denotes the $i^{th}$ included study datapoint, $se_{i}^{2}$ is the standard error from the $i^{th}$ included study estimate, and $\hat{\tau_{DL}^{2}}$ is the DL estimated random effects variance component, as defined in $(d)$ above.

Using a random effects model with inverse variance method and DL estimator, the estimate for the pooled effect size is then given as follows:

$$\hat{\theta}=\frac{\sum_{i} \hat{\theta}_{i}w_{i}^{\left\{ DL \right\}}}{\sum_{i} w_{i}^{\left\{ DL \right\}}} (f)$$

As previously mentioned, a log transformation is applied to the sampling data. In other words, we log transform IFR_2_ in the meta-analysis and appropriately back-transform the resulting point estimates and standard errors by exponentiating the log-transformed values. Hence, $\hat{\theta}$ in $\left( f \right)$above in this context is ${\hat{\theta}'}_{IFR_{2}}=log(\hat{\theta}_{IFR_{2}})$.

Then, to estimate the nationwide pooled infection fatality rate (IFR_2_) for India, countrywide IFR_2_ estimates (pre-calculated or computed) among included studies are pooled, as provided in $\left( f \right)$ above using the random effects framework detailed.

Using the *meta* package in R, pooled effect sizes are estimated, as well as 95% confidence intervals, following the detailed methodological framework.

**Methods D in S1 Text.** Risk of bias assessment among included articles

Using the Joanna Briggs Institute (JBI) tool, ***S1 Table*** contains the results from the risk of bias assessment across the included studies in the meta-analysis. Responses to each question in the JBI approach, as well as the cumulative score and rank of risk of bias, are detailed for each of the 15 included studies as well as the 1 nationwide serosurvey used to obtain meta-analyzable IFR estimates. The majority of the studies (12/15 included studies) received a perfect score. Two studies received a score of 7/8, maintaining a low risk of bias, and one study received a score of 5/8, with a moderate risk of bias.

**Methods E in S1 Text.** Assessment of publication bias

To formally test for funnel plot asymmetry, the Egger’s test is performed with a resulting p-value of 0.129. Since the significance level of the funnel plot intercept in the Egger’s test does not meet the benchmark of 0.05, we further check this result by conducting the Begg’s test (i.e., rank correlation test) and with a p-value of 0.715, we fail to reject the null and conclude that there appears to be no indication of publication bias. The results of these statistical tests aside, we do not expect that publication bias is of considerable concern for the following reasons. Firstly, seroprevalence studies at-large bely the included studies, which are inherently large studies with rigorous study designs, and thereby lead toward high precision and in turn low standard errors. Secondly, heterogeneity in the true effect size presented among the included studies for various reasons, including varying starting time points (although estimates are required to extend through June 2021, as encompassing the majority of the second wave in India), may attribute to the horizontal dispersion of the standard errors in ***Fig B in S1 Text***, wherein funnel plots operate under the assumption of a single true effect size.

**Table A in S1 Text.** PRISMA checklist [3]

| **Section and Topic** | **Item #** | **Checklist item** | **Location where item is reported** |
| --- | --- | --- | --- |
| **TITLE** | | | |
| Title | 1 | Identify the report as a systematic review. | Title Page |
| **ABSTRACT** | | | |
| Abstract | 2 | Background: Provide an explicit statement of the main objective(s) or question(s) the review addresses. Methods: Specify the inclusion and exclusion criteria for the review. Specify the information sources (e.g., databases, registers) used to identify studies and the date when each was last searched. Specify the methods used to assess risk of bias in the included studies. Specify the methods used to present and synthesise results. Results: Give the total number of included studies and participants and summarise relevant characteristics of studies. Present results for main outcomes, preferably indicating the number of included studies and participants for each. If meta-analysis was done, report the summary estimate and confidence/credible interval. If comparing groups, indicate the direction of the effect (i.e. which group is favoured). Discussion: Provide a brief summary of the limitations of the evidence included in the review (e.g., study risk of bias, inconsistency and imprecision). Provide a general interpretation of the results and important implications. Other: Specify the primary source of funding for the review. Provide the register name and registration number. | Title Page |
| **INTRODUCTION** | | | |
| Rationale | 3 | Describe the rationale for the review in the context of existing knowledge. | 2-3 |
| Objectives | 4 | Provide an explicit statement of the objective(s) or question(s) the review addresses. | 2-3 |
| **METHODS** | | | |
| Eligibility criteria | 5 | Specify the inclusion and exclusion criteria for the review and how studies were grouped for the syntheses. | 3, Fig 1 |
| Information sources | 6 | Specify all databases, registers, websites, organisations, reference lists and other sources searched or consulted to identify studies. Specify the date when each source was last searched or consulted. | 3, Methods A in S1 Text |
| Search strategy | 7 | Present the full search strategies for all databases, registers and websites, including any filters and limits used. | App B from [1], Methods A in S1 Text |
| Selection process | 8 | Specify the methods used to decide whether a study met the inclusion criteria of the review, including how many reviewers screened each record and each report retrieved, whether they worked independently, and if applicable, details of automation tools used in the process. | 3, App B from [1], Methods A in S1 Text |
| Data collection process | 9 | Specify the methods used to collect data from reports, including how many reviewers collected data from each report, whether they worked independently, any processes for obtaining or confirming data from study investigators, and if applicable, details of automation tools used in the process. | 3, App B from [1], Methods A in S1 Text |
| Data items | 10a | List and define all outcomes for which data were sought. Specify whether all results that were compatible with each outcome domain in each study were sought (e.g., for all measures, time points, analyses), and if not, the methods used to decide which results to collect. | 3-4, App B from [1], Methods A in S1 Text, Fig 1 |
|  | 10b | List and define all other variables for which data were sought (e.g., participant and intervention characteristics, funding sources). Describe any assumptions made about any missing or unclear information. | Table 1, Methods A and B in S1 Text, Fig A in S1 Text |
| Study risk of bias assessment | 11 | Specify the methods used to assess risk of bias in the included studies, including details of the tool(s) used, how many reviewers assessed each study and whether they worked independently, and if applicable, details of automation tools used in the process. | 5, Methods D in S1 Text |
| Effect measures | 12 | Specify for each outcome the effect measure(s) (e.g., risk ratio, mean difference) used in the synthesis or presentation of results. | 3-4, Methods B and C in S1 Text, Fig A in S1 Text |
| Synthesis methods | 13a | Describe the processes used to decide which studies were eligible for each synthesis (e.g., tabulating the study intervention characteristics and comparing against the planned groups for each synthesis (item #5)). | 3-4, App F and App G from [1], Table 1 |
|  | 13b | Describe any methods required to prepare the data for presentation or synthesis, such as handling of missing summary statistics, or data conversions. | Methods B and C in S1 Text, Fig B in S1 Text, App F and App G from [1] |
|  | 13c | Describe any methods used to tabulate or visually display results of individual studies and syntheses. | 3-4, Methods B and C in S1 Text |
|  | 13d | Describe any methods used to synthesize results and provide a rationale for the choice(s). If meta-analysis was performed, describe the model(s), method(s) to identify the presence and extent of statistical heterogeneity, and software package(s) used. | 3-4, Methods B and C in S1 Text |
|  | 13e | Describe any methods used to explore possible causes of heterogeneity among study results (e.g., subgroup analysis, meta-regression). | 3-4, Methods B and C in S1 Text |
|  | 13f | Describe any sensitivity analyses conducted to assess robustness of the synthesized results. | 5, Methods D and E in S1 Text |
| Reporting bias assessment | 14 | Describe any methods used to assess risk of bias due to missing results in a synthesis (arising from reporting biases). | Methods D and E in S1 Text |
| Certainty assessment | 15 | Describe any methods used to assess certainty (or confidence) in the body of evidence for an outcome. | 3-4, Methods B in S1 Text |
| **RESULTS** | | | |
| Study selection | 16a | Describe the results of the search and selection process, from the number of records identified in the search to the number of studies included in the review, ideally using a flow diagram. | 4, Fig 1 |
|  | 16b | Cite studies that might appear to meet the inclusion criteria, but which were excluded, and explain why they were excluded. | Fig 1, Table A in S1 Text |
| Study characteristics | 17 | Cite each included study and present its characteristics. | Fig 1, Table 1, App C from [1] |
| Risk of bias in studies | 18 | Present assessments of risk of bias for each included study. | 5, Methods D in S1 Text, S1 Table |
| Results of individual studies | 19 | For all outcomes, present, for each study: (a) summary statistics for each group (where appropriate) and (b) an effect estimate and its precision (e.g. confidence/credible interval), ideally using structured tables or plots. | 4-5, Table 1, Fig 2 |
| Results of syntheses | 20a | For each synthesis, briefly summarise the characteristics and risk of bias among contributing studies. | 5, Methods D in S1 Text |
|  | 20b | Present results of all statistical syntheses conducted. If meta-analysis was done, present for each the summary estimate and its precision (e.g. confidence/credible interval) and measures of statistical heterogeneity. If comparing groups, describe the direction of the effect. | 4-5, Fig 2 |
|  | 20c | Present results of all investigations of possible causes of heterogeneity among study results. | 5, Fig S2, Methods E in S1 Text |
|  | 20d | Present results of all sensitivity analyses conducted to assess the robustness of the synthesized results. | 5, Fig B in S1 Text |
| Reporting biases | 21 | Present assessments of risk of bias due to missing results (arising from reporting biases) for each synthesis assessed. | Methods E in S1 Text, Fig B in S1 Text |
| Certainty of evidence | 22 | Present assessments of certainty (or confidence) in the body of evidence for each outcome assessed. | 4-5, Fig 2, Methods E in S1 Text, Fig B in S1 Text |
| **DISCUSSION** | | | |
| Discussion | 23a | Provide a general interpretation of the results in the context of other evidence. | 5-6 |
|  | 23b | Discuss any limitations of the evidence included in the review. | 6-7 |
|  | 23c | Discuss any limitations of the review processes used. | 6-7 |
|  | 23d | Discuss implications of the results for practice, policy, and future research. | 7-8 |
| **OTHER INFORMATION** | | |  |
| Registration and protocol | 24a | Provide registration information for the review, including register name and registration number, or state that the review was not registered. | NA |
|  | 24b | Indicate where the review protocol can be accessed, or state that a protocol was not prepared. | NA |
|  | 24c | Describe and explain any amendments to information provided at registration or in the protocol. | NA |
| Support | 25 | Describe sources of financial or non-financial support for the review, and the role of the funders or sponsors in the review. | Title Page |
| Competing interests | 26 | Declare any competing interests of review authors. | Title Page |
| Availability of data, code and other materials | 27 | Report which of the following are publicly available and where they can be found: template data collection forms; data extracted from included studies; data used for all analyses; analytic code; any other materials used in the review. | 5 |

*NA = not applicable.*

**Table B in S1 Text.** Summary of regional mortality data from subnational studies in India from 2020-2021

| **Study** | **Time Period** | **Estimated Total Deaths (LL, UL) in Thousands** | **Under Reporting Factor (LL, UL)** | **Data Source(s)** | **Infection Fatality**  **Rate (%)** |
| --- | --- | --- | --- | --- | --- |
| ***Excess Deaths Studies*** | | | | | |
| Acosta et al., 2021  [4] | Mar '20-Apr '21 | 16 | *N/A* | Wall of Grief Database, Gujarat Death Registry | *N/A* |
| Bamezai et al., 2021  [5] | Apr-Jun 2021 | 300 | *N/A* | Private survey-based extrapolation for Bihar | *N/A* |
| Lewnard et al., 2022  [6] | Jan-Jun 2021 | *N/A* | 4 | CRS for Chennai | *N/A* |
| Banaji, 2021  [7] | Nov 2020 | *N/A* | *N/A* | Reported cases and deaths from covid19india.org, seroprevalence and excess deaths from Chennai, Tamil Nadu | 0.22-0.32 |
| Banaji, 2021  [8] | Jun-Jul 2020 | *N/A* | *N/A* | Reported deaths, seroprevalence and excess deaths from Mumbai,  Maharashtra | 0.28-0.40 |
| Cai et al., 2021  [9] | Jun-Aug 2020 (Karn.)  Jun-Jul 2020 (Mumb.) | *N/A* | *N/A* | Serosurvey and administrative data for Karnataka and Mumbai | *Karnataka*  Male age 10-49: 0.009 (0.007, 0.010)  Male age 50-89: 0.120 (0.090, 0.150)  Female age 10-49: 0.004 (0.004, 0.005)  Female age 50-89: 0.056 (0.043, 0.069)  *Mumbai*  Male age 10-49: 0.033 (0.032, 0.034)  Male age 50-89: 0.530 (0.516, 0.544)  Female age 10-49: 0.016 (0.016, 0.017)  Female age 50-89: 0.285 (0.277, 0.293) |
| ***Disease Transmission-based Studies*** | | | | | |
| ***Using Reported and Unreported COVID-19 Deaths*** | | | | | |
| Campbell & Gustafson, 2021  [10] | May-Jun 2020 | *N/A* | *N/A* | Reported deaths from ourworldindata.org/ coronavirus/country/india, COVID-19 infections from Delhi serosurvey,  death underreporting factor from Purkayastha et al. (2021) | *Delhi*  Chen et al.-based: 0.12 (0.07, 0.34)  Serotracker-based: 0.18 (0.07, 0.43) |
| **Study** | **Time Period** | **Estimated Total Deaths (LL, UL) in Thousands** | **Under Reporting Factor (LL, UL)** | **Data Source(s)** | **Infection Fatality**  **Rate (%)** |
| Bhaduri et al., 2022  [11] | Jul 2021 | Delhi: 72.4 (69.6, 75.1)  Mumbai: 43.9 (41.9, 46.3) | Delhi: 6.29 (6.24, 6.33)  Mumbai: 3.86 (3.69, 4.08) | Reported deaths and cases from covid19india.org for Delhi and Mumbai | *N/A* |
| Goli & James, 2020  [12] | Apr 2020 | *N/A* | *N/A* | State-specific reported deaths and cases from covid19india.org, testing information from ICMR, age distributions from 2011 census, IFR for China from  Verity et al. (2020) | Andhra Pradesh:  Delhi: 0.396  Gujarat: 0.437  Haryana: 0.394  Jammu & Kashmir: 0.358  Karnataka: 0.440  Kerala: 0.579  Madhya Pradesh: 0.364  Maharashtra: 0.435  Rajasthan: 0.358  Tamil Nadu: 0.524  Telangana: 0.432  Uttar Pradesh: 0.335 |
| Goult et al., 2022  [13] | Jul 2020 | *N/A* | *N/A* | Reported deaths and cases for Kerala | 0.4 |
| ***Using Reported COVID-19 Deaths*** | | | | | |
| Hazra et al., 2021  [14] | Mar ‘20–Feb ‘21 | *N/A* | Karnataka: 2.2 (range: 2-5) | State-specific reported deaths and cases from covid19india.org, age distribution from 2011 census | Bengaluru: 0.05  Chennai: 0.052  Delhi: 0.10  Mumbai: 0.15  Pune: 0.17 |
| ***COVID-19 surveillance-based studies*** | | | | | |
| George et al., 2022  [15] | Jan ’20-Jul ‘21 | *N/A* | *N/A* | Bangalore rural district household survey | 0.016 |
| Laxminarayan et al., 2021  [16] | May-Oct 2020 | *N/A* | *N/A* | Cross-sectional survey in Madurai, Tamil Nadu | 0.043 |
| Sharma et al., 2021  [17] | Aug, Sep, Oct 2020 | *N/A* | *N/A* | Repeated cross-sectional serosurvey in Delhi | Aug: 0.77 (0.75–0.79) to 0.79 (0.76–0.81)  Sep: 0.98 (0.95–1.01) to 1.03 (1.00–1.06)  Oct: 1.27 (1.24–1.31) to 1.34 (1.31–1.38) |
| Malani et al., 2021  [18] | Jun-Jul 2020 | *N/A* | *N/A* | Cross-sectional serosurvey in Mumbai, Maharashtra | Slum: 0.076  Non-slum: 0.263 |
| Babu et al., 2021  [19] | Sep 2020 | *N/A* | *N/A* | Cross-sectional serosurvey in Karnataka | 0.05 |
| **Study** | **Time Period** | **Estimated Total Deaths (LL, UL) in Thousands** | **Under Reporting Factor (LL, UL)** | **Data Source(s)** | **Infection Fatality**  **Rate (%)** |
| Banerjee et al., 2021  [20] | Oct 2020 | *N/A* | *N/A* | Cross-sectional serosurvey in Pimpri-Chinchiwad, Maharashtra | 0.17 |
| Malani et al., 2021  [21] | Oct-Nov 2020 | *N/A* | *N/A* | Cross-sectional serosurvey in Tamil Nadu | Female age 18–29: 0.002  Female age 30–39: 0.006  Female age 40–49: 0.019  Female age 50–59: 0.060  Female age 60–69: 0.143  Female age ≥70: 0.266  Male age 18–29: 0.003  Male age 30–39: 0.015  Male age 40–49: 0.045  Male age 50–59: 0.164  Male age 60–69: 0.380  Male age ≥70:  0.923 |

*Notes:* N/A=Not available, CRS=Civil Registration System, ICMR=Indian Council for Medical Research. All estimates in this table are as

directly reported in the cited study. Lower and upper uncertainty bounds for IFR estimates, as well as for Underreporting Factors, are included

in this table, when provided in the study.

**Fig A in S1 Text.** Description of data extraction

1. From meta-analyzed excess deaths studies

**Data Extraction: Numerator of IFR_2_**

**Excess deaths studies, through at least June 2021 and**

**up to Dec 2021:**

Estimated deaths of $\hat{d} (95\% CI:)$

**Total Cumulative Deaths**

**IFR_2_ (%)^c^ = (Total Cumulative Deaths ÷ Total Cumulative Infections) × 100**

**India’s 4^th^ nationwide serosurvey (14 June-6 July 2021):**

Estimated COVID-19 prevalence of 67.7% (95% CI: 66.4, 68.7) for ages ≥ 6 years

**World Bank 2019 projection for India:**

Population estimate of 1.366 billion persons

**2011 census age composition for India:**

Proportion of population aged ≥ 6 years of 82.8%

**Data Extraction: Denominator of IFR_2_**

**Total Cumulative Infections^b^**

1. From disease transmission-based studies, not meta-analyzed (insufficient number of identified studies) without directly provided IFR_2_

**Disease transmission-based studies, through at least June 2021 and**

**up to Dec 2021:**

Estimated deaths of $\hat{d} (95\% CI:)$

**Data Extraction: Numerator of IFR_2_**

**Total Cumulative Deaths**

**IFR_2_ (%)^c^ = (Total Cumulative Deaths ÷ Total Cumulative Infections) × 100**

**India’s 4^th^ nationwide serosurvey^a^ (14 June-6 July 2021):**

Estimated COVID-19 prevalence of 67.7% (95% CI: 66.4, 68.7) for ages ≥ 6 years

**World Bank 2019 projection for India:**

Population estimate of 1.366 billion persons

**2011 census age composition for India:**

Proportion of population aged ≥ 6 years of 82.8%

**Data Extraction: Denominator of IFR_2_**

**Total Cumulative Infections^b^**

1. From disease transmission-based studies, not meta-analyzed (insufficient number of identified studies) with directly provided IFR_2_

**Disease transmission-based studies, through at least June 2021 and**

**up to Dec 2021:**

Estimated infection fatality rate, when provided of

$$\hat{IFR}_{2} (95\% CI:)$$

**IFR_2_ (%) (95% CI:)**

**Data Extraction: IFR_2_**

a. Seroprevalence estimate in this figure are as directly reported in the 4^th^ nationwide serosurvey [2].

b. Total Cumulative Infections is computed as follows. First, an age-adjusted population is obtained through multiplying the 2019 population estimate for India of 1.366 billion from the World Bank (https://data.worldbank.org/indicator/SP.POP.TOTL?locations=IN&most_recent_year_

desc=false) by the proportion of the population aged ≥ 6 years old, as obtained from the 2011 census age composition (https://censusindia.gov.in/vital_statistics/SRS_Report/9Chap%202%20-%202011.pdf). Then, Total Cumulative Infections is computed as the age-adjusted population multiplied by the seroprevalence estimate of 67.6% among those aged ≥ 6 years old, from the 4^th^ nationwide serosurvey for India [2].

c. Details and formulas regarding calculations for 95% CI for IFR_2_ are contained in **Methods B in S1 Text**.

**Fig B in S1 Text.** Funnel plot for assessment of publication bias among included studies

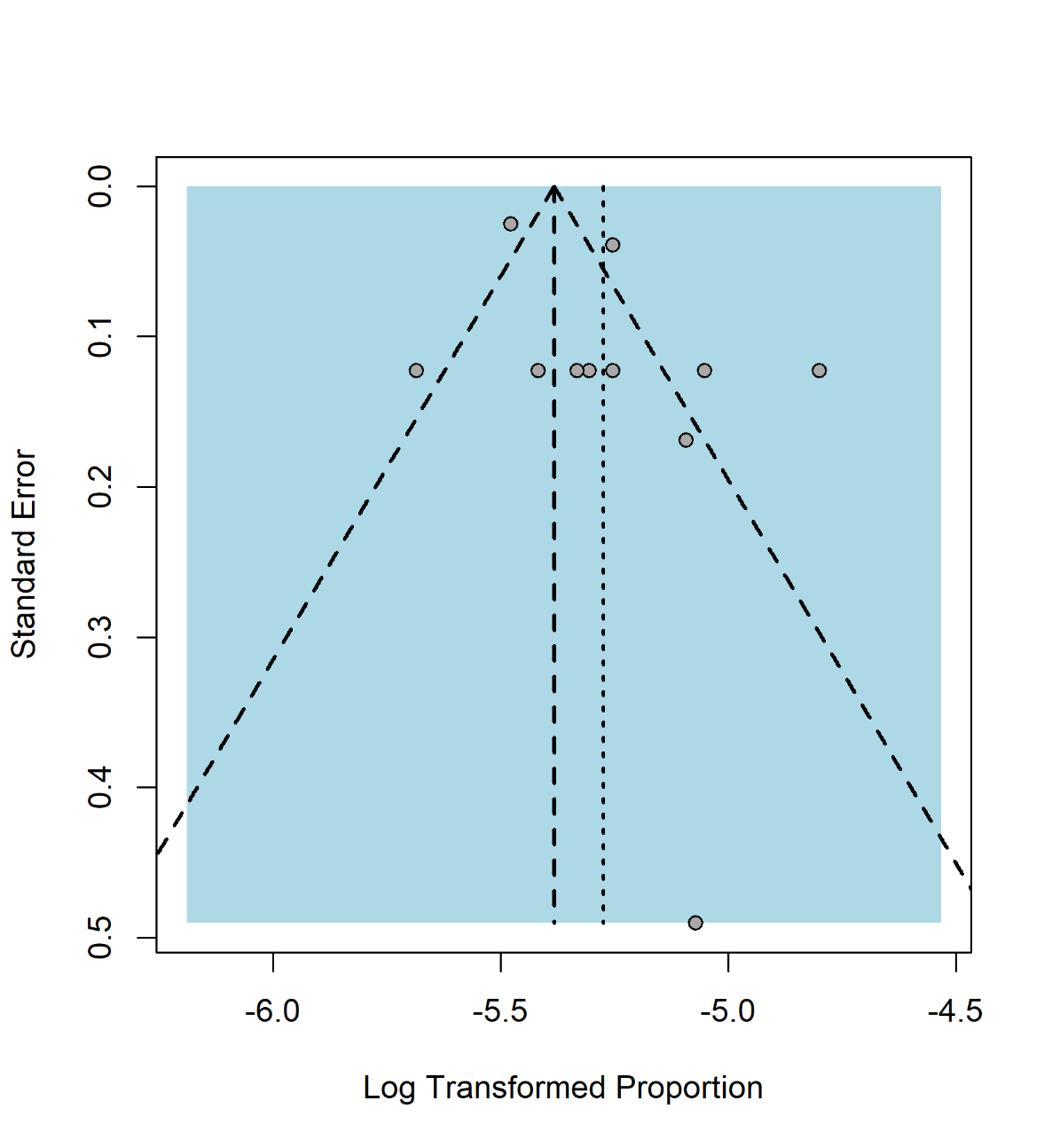


**References**

1. Zimmermann L, Bhattacharya S, Purkayastha S, Kundu R, Bhaduri R, Ghosh P, et al. SARS-CoV-2 infection fatality rates in India: systematic review, meta-analysis and model-based estimation. 2021 Sep p. 2021.09.08.21263296. doi:10.1101/2021.09.08.21263296

2. Murhekar MV, Bhatnagar T, Thangaraj JWV, Saravanakumar V, Kumar MS, Selvaraju S, et al. Seroprevalence of IgG antibodies against SARS-CoV-2 among the general population and healthcare workers in India, June–July 2021: A population-based cross-sectional study. PLOS Med. 2021;18: e1003877. doi:10.1371/journal.pmed.1003877

3. Page MJ, McKenzie JE, Bossuyt PM, Boutron I, Hoffmann TC, Mulrow CD, et al. The PRISMA 2020 statement: an updated guideline for reporting systematic reviews. Syst Rev. 2021;10: 89. doi:10.1186/s13643-021-01626-4

4. Acosta RJ, Patnaik B, Buckee C, Kiang MV, Irizarry RA, Balsari S, et al. All-cause excess mortality in the State of Gujarat, India, during the COVID-19 pandemic (March 2020-April 2021). medRxiv; 2022. p. 2021.08.22.21262432. doi:10.1101/2021.08.22.21262432

5. Bamezai A, Banaji M, Gupta A, Pandey S, Mr S, Sharma K, et al. Survey evidence of excess mortality in Bihar in the second COVID-19 surge. SocArXiv; 2021. doi:10.31235/osf.io/zxq97

6. Lewnard JA, Mahmud A, Narayan T, Wahl B, Selvavinayagam TS, B CM, et al. All-cause mortality during the COVID-19 pandemic in Chennai, India: an observational study. Lancet Infect Dis. 2022;22: 463–472. doi:10.1016/S1473-3099(21)00746-5

7. Banaji M. Estimates of COVID-19 infection fatality rate in Chennai - Mathematics. 2021 [cited 26 May 2022]. Available: https://maths.mdx.ac.uk/research/modelling-the-covid-19-pandemic/chennaiifrestimates/

8. Banaji M. Estimating COVID-19 infection fatality rate in Mumbai during 2020. medRxiv. 2021; 2021.04.08.21255101. doi:10.1101/2021.04.08.21255101

9. Cai R, Novosad P, Tandel V, Asher S, Malani A. Representative estimates of COVID-19 infection fatality rates from four locations in India: cross-sectional study. BMJ Open. 2021;11: e050920. doi:10.1136/bmjopen-2021-050920

10. Campbell H, Gustafson P. Inferring the COVID-19 infection fatality rate in the community-dwelling population: a simple Bayesian evidence synthesis of seroprevalence study data and imprecise mortality data. Epidemiol Infect. 2021;149. doi:10.1017/S0950268821002405

11. Bhaduri R, Kundu R, Purkayastha S, Kleinsasser M, Beesley LJ, Mukherjee B, et al. Extending the susceptible‐exposed‐infected‐removed (SEIR) model to handle the false negative rate and symptom‐based administration of COVID‐19 diagnostic tests: SEIR‐fansy. Stat Med. 2022;41: 2317–2337. doi:10.1002/sim.9357

12. Goli S, James KS. How much of SARS-CoV-2 Infections is India detecting? A model-based estimation. medRxiv. 2020; 2020.04.09.20059014. doi:10.1101/2020.04.09.20059014

13. Goult E, Sathyendranath S, Kovač Ž, Kong CE, Stipanović P, Abdulaziz A, et al. Analysis of non-pharmaceutical interventions and their impacts on COVID-19 in Kerala. Sci Rep. 2022;12: 584. doi:10.1038/s41598-021-04488-x

14. Hazra DK, Pujari BS, Shekatkar SM, Mozaffer F, Sinha S, Guttal V, et al. The INDSCI-SIM model for COVID-19 in India. medRxiv. 2021; 2021.06.02.21258203. doi:10.1101/2021.06.02.21258203

15. George CE, Inbaraj LR, Rajukutty S, Joan RF, Suseeladevi AK, Muthuraj S, et al. Seroprevalence of COVID-19 infection among vaccine naïve population after the second surge (June 2020) in a rural district of South India: A community-based cross-sectional study. PLOS ONE. 2022;17: e0265236. doi:10.1371/journal.pone.0265236

16. Laxminarayan R, B CM, G VT, Kumar KVA, Wahl B, Lewnard JA. SARS-CoV-2 infection and mortality during the first epidemic wave in Madurai, south India: a prospective, active surveillance study. Lancet Infect Dis. 2021;21: 1665–1676. doi:10.1016/S1473-3099(21)00393-5

17. Sharma P, Chawla R, Bakshi R, Saxena S, Basu S, Bharti PK, et al. Seroprevalence of antibodies to SARS-CoV-2 and predictors of seropositivity among employees of a teaching hospital in New Delhi, India. Osong Public Health Res Perspect. 2021;12: 88–95. doi:10.24171/j.phrp.2021.12.2.06

18. Malani A, Shah D, Kang G, Lobo GN, Shastri J, Mohanan M, et al. Seroprevalence of SARS-CoV-2 in slums versus non-slums in Mumbai, India. Lancet Glob Health. 2021;9: e110–e111. doi:10.1016/S2214-109X(20)30467-8

19. Babu GR, Sundaresan R, Athreya S, Akhtar J, Pandey PK, Maroor PS, et al. The burden of active infection and anti-SARS-CoV-2 IgG antibodies in the general population: Results from a statewide sentinel-based population survey in Karnataka, India. Int J Infect Dis. 2021;108: 27–36. doi:10.1016/j.ijid.2021.05.043

20. Banerjee A, Gaikwad B, Desale A, Jadhav SL, Rathod H, Srivastava K. Severe acute respiratory syndrome-coronavirus-2 seroprevalence study in Pimpri-Chinchwad, Maharashtra, India coinciding with falling trend – Do the results suggest imminent herd immunity? Indian J Public Health. 2021;65: 256. doi:10.4103/ijph.IJPH_122_21

21. Malani A, Ramachandran S, Tandel V, Parasa R, Imad S, Sudharshini S, et al. SARS-CoV-2 Seroprevalence in Tamil Nadu in October-November 2020. medRxiv. 2021; 2021.02.03.21250949. doi:10.1101/2021.02.03.21250949
