# Supplementary material for: Meta-analysis of nationwide SARS-CoV-2 infection fatality rates in India
Source: PLOS Glob Public Health. 2022 Sep 19;2(9):e0000897. doi: 10.1371/journal.pgph.0000897 (PMC10021252; doi:10.1371/journal.pgph.0000897)
Supplement: Attachment — Submitted filename: Reviewer_Response_Letter_PLOSGPH.docx [file pgph.0000897.s006.docx]

**Response to Reviewers: PGPH-D-21-01072:** **Meta-analysis of nationwide SARS-CoV-2 infection fatality rates in India**

Authors:

Lauren Zimmermann MS^1,2^, Bhramar Mukherjee PhD^1,2,3^

Affiliations:

^1^ Department of Biostatistics, University of Michigan, Ann Arbor, MI, USA

^2^ Center for Precision Health Data Science, University of Michigan, Ann Arbor, MI, USA

^3^ Department of Epidemiology, University of Michigan, Ann Arbor, MI, USA

* Correspondence to:

Lauren Zimmermann

Department of Biostatistics

University of Michigan School of Public Health

1415 Washington Heights

Ann Arbor, MI 48109.

lzimm@umich.edu

We would like to thank the review panel for their constructive feedback that has led to a substantial improvement in the previously submitted version of the manuscript. In the following document, we provide an itemized response to each reviewer comment. In addition to addressing comments from reviewers, we also updated the studies included in our meta-analysis to reflect the most recently available publications and preprints through May 26, 2022. We now include 5 more studies as a result in our updated review for a total of 15 studies, from which 11 estimates were included in the quantitative synthesis. The methods by which we updated our search are detailed in the responses to reviewers below, in the cover letter accompanying this submission, and in our manuscript. We hope the revised manuscript is found suitable for publication in *PLOS Global Public Health*. Thank you for allowing us the opportunity to revise our manuscript and to address the reviewer comments.

=====================

**Reviewer #1 comments:**

The paper is potentially a valuable addition to the literature on the impact of the pandemic in LMICs. In a context of hugely variable surveillance and major gaps in our knowledge of the impact of the pandemic in the Global South, such work is of high importance. Given India's sheer size and weak surveillance, understanding pandemic mortality in India is perhaps the most critical challenge to understanding pandemic mortality worldwide.

However, there are some limitations to the work, which the authors would do well to address.

**Response:** We thank the reviewer for the positive comments.

R1.1 General Comments: It would be helpful if the 15 studies which were included in the meta-analysis are listed clearly, perhaps in a table, along with an outline of what kind of data or analysis appears in each, and how this data is used to arrive at IFR estimates.

**Response:** We thank the reviewer for the comments. We changed Table 1 to separately list the 15 studies that were qualitatively synthesized, and the 9 studies comprising 11 datapoints included in the meta-analysis as well as the underlying data sources used within each included study (see updated Table 1 below).

We have also newly included in the supplementary materials Fig S1, that is a flow diagram showing how we use data from the included studies to derive IFR estimates, which we subsequently meta-analyze (see Fig S1 below). We reference this table in the methods section on lines 108-110 as follows: “*A detailed explanation of the meta-analysis framework is provided in the* ***M******ethods S2*** *and* ***Methods S3****, including* ***Fig S1*** *displaying the process from data extraction to obtaining meta-analyzable IFRs.*”

*Notes:* Asterisk (*****) denotes that the excess deaths study was included in the quantitative meta-analysis, being through at least June 2021.

N/A=Not available, CRS=Civil Registration System, MLA=Member of the Legislative Assembly sample, CVoter=CVoter India Omnibus

telephone survey, HMIS=Health Management Information System, ACM=all-cause mortality, CMIE=Center for Monitoring Indian

Economy Consumer Pyramids Household survey, IDSP=Integrated Disease Surveillance Programme (for Goa, India). JHU

CSSE=Johns Hopkins University Center for Systems Science and Engineering, MCCD=Medical Certification of Cause of Death from

Ministry of Home Affairs, WDI=World Bank’s World Development Indicators. Lower and upper uncertainty bounds for all-cause excess

deaths estimates are included in this table, when provided in the study.

[a] Estimated total cumulative infections is calculated as the seroprevalence of 67.6% among ages ≥ 6 years from the latest 4^th^ nationwide

serosurvey study in India (*6*) multiplied by the age-adjusted population (additional details are included in Methods S2 and Fig S1).

[1] COVID-19 Reported Deaths are obtained from covid19india.org, unless otherwise noted.

[2] Underreporting Factor is computed as Excess Deaths divided by COVID-19 Reported Deaths, unless otherwise noted.

[3] Underreporting Factor (URF), as well as COVID-19 Reported Deaths are directly reported in this study. Hence, the URF in this table

is the precalculated estimate provided.

[4] Excess Deaths, as well as COVID-19 Reported Deaths, are directly reported in this study.

[5] The COVID-19 Reported Deaths provided in this study are across select states in the Civil Registration System (CRS).

[6] The precalculated Underreporting Factor and COVID-19 Reported Deaths reported in this study are through September 2021.

[7] Numerical estimates for total deaths are unavailable for Rahmandad et al. (2021) (*17*), and are thereby displayed as not available

in this table.

R1.2 General Comments: The eligibility criteria for included and excluded papers (Figure 2) are not clear - it appears that papers which either model pandemic mortality or include prevalence or mortality data were included. Were papers focussing on subnational data excluded, or were no such papers found to meet the eligibility criteria? In sum, the framework needs to be spelled out more clearly - Figure 2 does not seem adequate.

**Response:** We thank the reviewer for the recommendation. Yes we include only estimates with a nationwide mortality or prevalence estimate, and as such subnational studies are excluded in the meta-analysis. We have included further clarification on our exclusion of subnational studies through adding the following sentence in the methods section on lines 106-107: “*While this meta-analysis focuses on nationwide studies in India, we summarize the* ***18*** *other* *subnational/regional studies (not meta-analyzed) in* ***Table S2***”. As such, we agree there are numerous subnational studies that ought to be recognized, and we have newly added to the supplementary materials Table S2 that has subnational data from excess deaths and model-based studies organized by region, which we do not meta-analyze. This table in the supplementary materials is shown below.

**Table S2.** Summary of regional mortality data from subnational studies in India from 2020-2021

| **Study** | **Time Period** | **Estimated Total Deaths (LL, UL) in Thousands** | **Under Reporting Factor (LL, UL)** | **Data Source(s)** | **Infection Fatality**  **Rate (%)** |
| --- | --- | --- | --- | --- | --- |
| ***Excess Deaths Studies*** | | | | | |
| Acosta et al., 2021  [3] | Mar '20-Apr '21 | 16 | *N/A* | Wall of Grief Database, Gujarat Death Registry | *N/A* |
| Bamezai et al., 2021  [4] | Apr-Jun 2021 | 300 | *N/A* | Private survey-based extrapolation for Bihar | *N/A* |
| Lewnard et al., 2021  [5] | Jan-Jun 2021 | *N/A* | 4 | CRS for Chennai | *N/A* |
| Banaji, 2021  [6] | Nov 2020 | *N/A* | *N/A* | Reported cases and deaths from covid19india.org, seroprevalence and excess deaths from Chennai, Tamil Nadu | 0.22-0.32 |
| Banaji, 2021  [7] | Jun-Jul 2020 | *N/A* | *N/A* | Reported deaths, seroprevalence and excess deaths from Mumbai,  Maharashtra | 0.28-0.40 |
| Cai et al., 2021  [8] | Jun-Aug 2020 (Karn.)  Jun-Jul 2020 (Mumb.) | *N/A* | *N/A* | Serosurvey and administrative data for Karnataka and Mumbai | *Karnataka*  Male age 10-49: 0.009 (0.007, 0.010)  Male age 50-89: 0.120 (0.090, 0.150)  Female age 10-49: 0.004 (0.004, 0.005)  Female age 50-89: 0.056 (0.043, 0.069)  *Mumbai*  Male age 10-49: 0.033 (0.032, 0.034)  Male age 50-89: 0.530 (0.516, 0.544)  Female age 10-49: 0.016 (0.016, 0.017)  Female age 50-89: 0.285 (0.277, 0.293) |
| ***Disease Transmission-based Studies*** | | | | | |
| ***Using Reported and Unreported COVID-19 Deaths*** | | | | | |
| Campbell & Gustafson, 2021  [9] | May-Jun 2020 | *N/A* | *N/A* | Reported deaths from ourworldindata.org/ coronavirus/country/india, COVID-19 infections from Delhi serosurvey,  death underreporting factor from Purkayastha et al. (2021) | *Delhi*  Chen et al.-based: 0.12 (0.07, 0.34)  Serotracker-based: 0.18 (0.07, 0.43) |
| **Study** | **Time Period** | **Estimated Total Deaths (LL, UL) in Thousands** | **Under Reporting Factor (LL, UL)** | **Data Source(s)** | **Infection Fatality**  **Rate (%)** |
| Bhaduri et al., 2022  [10] | Jul 2021 | Delhi: 72.4 (69.6, 75.1)  Mumbai: 43.9 (41.9, 46.3) | Delhi: 6.29 (6.24, 6.33)  Mumbai: 3.86 (3.69, 4.08) | Reported deaths and cases from covid19india.org for Delhi and Mumbai | *N/A* |
| Goli & James, 2020  [11] | Apr 2020 | *N/A* | *N/A* | State-specific reported deaths and cases from covid19india.org, testing information from ICMR, age distributions from 2011 census, IFR for China from  Verity et al. (2020) | Andhra Pradesh:  Delhi: 0.396  Gujarat: 0.437  Haryana: 0.394  Jammu & Kashmir: 0.358  Karnataka: 0.440  Kerala: 0.579  Madhya Pradesh: 0.364  Maharashtra: 0.435  Rajasthan: 0.358  Tamil Nadu: 0.524  Telangana: 0.432  Uttar Pradesh: 0.335 |
| Goult et al., 2022  [12] | Jul 2020 | *N/A* | *N/A* | Reported deaths and cases for Kerala | 0.4% |
| ***Using Reported COVID-19 Deaths*** | | | | | |
| Hazra et al., 2021  [13] | Mar ‘20–Feb ‘21 | *N/A* | Karnataka: 2.2 (range: 2-5) | State-specific reported deaths and cases from covid19india.org, age distribution from 2011 census | Bengaluru: 0.05  Chennai: 0.052  Delhi: 0.10  Mumbai: 0.15  Pune: 0.17 |
| ***COVID-19 surveillance-based studies*** | | | | | |
| George et al., 2022  [14] | Jan ’20-Jul ‘21 | *N/A* | *N/A* | Bangalore rural district household survey | 0.016 |
| Laxminarayan et al., 2022  [15] | May-Oct 2020 | *N/A* | *N/A* | Cross-sectional survey in Madurai, Tamil Nadu | 0.043 |
| Sharma et al., 2020  [16] | Aug, Sep, Oct 2020 | *N/A* | *N/A* | Repeated cross-sectional serosurvey in Delhi | Aug: 0.77 (0.75–0.79) to 0.79 (0.76–0.81)  Sep: 0.98 (0.95–1.01) to 1.03 (1.00–1.06)  Oct: 1.27 (1.24–1.31) to 1.34 (1.31–1.38) |
| Malani et al., 2021  [17] | Jun-Jul 2020 | *N/A* | *N/A* | Cross-sectional serosurvey in Mumbai, Maharashtra | Slum: 0.076  Non-slum: 0.263 |
| Babu et al., 2021  [18] | Sep 2020 | *N/A* | *N/A* | Cross-sectional serosurvey in Karnataka | 0.05 |
| **Study** | **Time Period** | **Estimated Total Deaths (LL, UL) in Thousands** | **Under Reporting Factor (LL, UL)** | **Data Source(s)** | **Infection Fatality**  **Rate (%)** |
| Banerjee et al., 2021  [19] | Oct 2020 | *N/A* | *N/A* | Cross-sectional serosurvey in Pimpri-Chinchiwad, Maharashtra | 0.17 |
| Malani et al., 2021  [20] | Oct-Nov 2020 | *N/A* | *N/A* | Cross-sectional serosurvey in Tamil Nadu | Female age 18–29: 0.002  Female age 30–39: 0.006  Female age 40–49: 0.019  Female age 50–59: 0.060  Female age 60–69: 0.143  Female age ≥70: 0.266  Male age 18–29: 0.003  Male age 30–39: 0.015  Male age 40–49: 0.045  Male age 50–59: 0.164  Male age 60–69: 0.380  Male age ≥70: 0.923% |

*Notes:* N/A=Not available, CRS=Civil Registration System, ICMR=Indian Council for Medical Research. All estimates in this table are as directly reported in the cited

study. Lower and upper uncertainty bounds for IFR estimates, as well as for Underreporting Factor estimates, are included in this table, when provided in the study.

R1.3 General Comments: In reading a meta-analysis there is often the assumption in the reader's mind - rightly or wrongly - that the studies are in some sense "independent". They may involve different data-sets, different geographical regions, different methodologies, etc. Here, however, it appears that many of the estimates are based around overlapping data and techniques. This applies for example to the estimates of excess mortality based on civil registration data, and also perhaps to estimates of prevalence based on serosurveys. Overall, it appears that there is relatively little "independent" data behind the numbers from different papers, increasing the risk of systematic bias (more on this below). This warrants at least some discussion.

**Response:** We thank the reviewer for this important remark. We agree that it is important to note that multiple studies rely on excess deaths derived from civil registration system (CRS) data or infections derived from the nationwide seroprevalence studies, as examples, which may bias the resulting estimates. Also, the updated meta-analysis results do not include datapoints from related or repeated studies. In the case of repeated studies, we use the latest version in the meta-analysis. We have included a few sentences on these critical points in the discussion section in lines 153-156: “*Second, multiple studies rely on excess deaths derived from common sources, such as the civil registration system (CRS) data, as well as infections derived from nationwide serosurveys, which rules out independence between included studies and may bias the resulting pooled estimate.*”

R1.4 General Comments: The study includes both data- and model-based estimates, and it is observed that the model-based estimates of IFR are more conservative than data-based ones. However, model-based estimates reflect a complex array of assumptions - including, perhaps, about ascertainment of infections and deaths, and even sometimes about IFR itself. This leads to the possibility of circularity. Without some discussion of the assumptions behind the models it is very hard to interpret model-based IFR estimates, or to know whether they deserve the same weight as data-based ones. (Although data-based estimates also rely on a host of assumptions, these are in general more obvious to see). This seems to me to be of critical importance in a meta-analysis which relies on a small number of papers, a sizeable fraction of which are modelling papers.

**Response:** We thank the reviewer for this notable consideration. We had intended to stratify by excess deaths and disease transmission-based studies, which is similar to the grouping in Table 1. However, an insufficient number of disease transmission-based studies rendered us unable to reliably estimate a pooled nationwide IFR separately among this study type (although we provide a pooled estimate for this study category for comparison purposes). Therefore, we now report one pooled nationwide IFRs, one across excess deaths studies only to suppress heterogeneity attributed to the study type. In turn, Fig 2 is updated to reflect this change of including excess deaths studies only. This updated Fig 2 is included below. We have included a few sentences on this important point in the methods and results sections (see manuscript excerpts below).

In the methods section at lines 93-101: “*Using this approach, 4,971 citations were screened resulting in* ***15*** *studies classified into the following three groups: excess deaths studies (9 articles), disease transmission-based studies estimating unreported deaths (5 articles), disease transmission-based studies using reported deaths only (1* *article). Since the three groups are not directly comparable, among the 15 studies, the* ***9*** *from excess deaths studies with* ***11*** *datapoints are included in the nationwide quantitative synthesis. We were unable to stratify and separately meta-analyze disease transmission-based estimates (less than 3 studies rendered through at least June 2021 in the search verification).*”

In the results section at lines 127-130: “*However, disease transmission-based studies give us a nationwide pooled IFR_2_ estimate of 0.34% (95% CI: 0.28% – 0.41%), although we caution that this second estimate relies on less than 3 data points.*”

**Fig 2. Nationwide estimated pooled IFR_2_ of SARS-CoV-2 for India, through June 2021 and extending to December 2021.**

Notes: Included studies listed in this forest plot are categorized as excess deaths studies. There were too few disease transmission-based studies (less than 3 studies through June 2021) identified by the search, and therefore, further meta-analyzing the additional category of disease transmission-based studies was not feasible.

R1.5 General Comments: On the theme of model-based estimates, disproportionate weight appears to be given in the text to the IHME modelling, whose estimates appear to be outliers even amongst the model-based estimates (and certainly amongst the estimates from all sources). Are the IHME projections available in any kind of preprint, perhaps submitted for peer-review? Is this work sufficiently transparent to meet the criteria for this kind of analysis? (I have some doubts about this, but these would be allayed if the authors referenced a technical document which included details of how the IHME estimates are arrived at, specifically for India.)

**Response:** We thank the reviewer for the comments. We agree that the IHME estimates being model-based and subject to other assumptions are outliers. We have revised the discussion section and have removed the previous Figure 3 from the manuscript. Instead, we tried to quote published estimates from the following global studies: WHO estimates, IHME estimates (Barber et al, 2022; Wang et al, 2022) and The Economist estimates in a more balanced way.

- Barber, R. M., Sorensen, R. J., Pigott, D. M., Bisignano, C., Carter, A., Amlag, J. O., ... & Murray, C. J. (2022). Estimating global, regional, and national daily and cumulative infections with SARS-CoV-2 through Nov 14, 2021: a statistical analysis. *The Lancet*.
- Wang, H., Paulson, K. R., Pease, S. A., Watson, S., Comfort, H., Zheng, P., ... & Murray, C. J. (2022). Estimating excess mortality due to the COVID-19 pandemic: a systematic analysis of COVID-19-related mortality, 2020–21. *The Lancet*.
- Knutson, V., Aleshin-Guendel, S., Karlinsky, A., Msemburi, W., & Wakefield, J. (2022). Estimating Global and Country-Specific Excess Mortality During the COVID-19 Pandemic. arXiv preprint arXiv:2205.09081. (WHO reference)
- The Economist, S. U. Solstad, The pandemic’s true death toll. *The Economist* (2021).

In the discussion section on lines 172-185: “*Considering the three countries with the highest cumulative reported deaths (as of December 31, 2021), namely, India, Brazil, and the United States (in ascending order), the IFR_2_ (as of 14 November 2021) reported by Barber et al. (2022) appears to be the lowest in India (IFR_2_ of 0.3%) compared to the US (IFR_2_ of 0.9%) and Brazil (IFR_2_ of 0.5%) (7). This is due to the very large number of estimated cumulative infections in India (approximately 1 billion, through mid-November 2021 (7)). With respect to the total number of deaths, Wang et al. (2022) estimate deaths to be underreported by a factor of 8.3, 1.3, and 1.2 for India, the US, and Brazil, respectively (5). This reconciles at-large with death underreporting factors reliant on WHO estimates (similarly through 31^st^ December 2021) of 9.8, 1.1, and 1.1 for India (4.7 million excess deaths and 481,080 reported deaths), the US (933,547 excess deaths and 818,464 reported deaths), and Brazil (681,514 excess deaths and 618,817 reported deaths), respectively (9). These rankings indicate that underreporting of deaths (through 31^st^ December 2021) is particularly acute for India.*”

R1.6 General Comments: Overall, potential systematic biases deserve much more discussion. For example, in estimates based on survey or civil registration data: the possibility of non-COVID excess mortality, particularly during periods when healthcare infrastructure is overwhelmed; potential sampling biases in survey-based estimates; issues around incompleteness of death registration and possible differential impact of the pandemic on communities where registration is higher/lower; possible issues with the fact that available data is often from sources (e.g. online systems) which are incomplete. Although a meta-analysis may not be the place to discuss in detail potential biases in each study, it is important for readers to understand the limitations to the work, which are often common to many papers.

**Response:** We thank the reviewer for these important considerations. We agree that the attribution of 100% of excess deaths to COVID-19 does not account for possible occurrence of non-COVID excess mortality during the pandemic time period, and we have included the below sentences in the manuscript on this point.

In the results section at lines 124-127: “*This estimate attributes 100% of excess deaths to SARS-CoV-2 during 2020-2021. In actuality, the proportion of excess deaths resulting from COVID-19 is not likely to wholly account for the total excess deaths during the pandemic period, and as such this estimate of 0.51% is likely an overestimate.*”

In the discussion section at lines 143-147: “*The disease transmission-based estimates qualitatively appear to be more conservative than the ones that originated from excess deaths studies. One possible explanation could stem from the fact that most of the excess death studies are based on all-cause-mortality data and do not quantify the proportion of the excess deaths attributable to COVID-19.*”

In light of other critical points the reviewer raises, we have also included the following sentences to our limitations in the discussion section in lines 156-167: “*India recently released the CRS data for 2020, but most studies estimate largest excess deaths during April-June of 2021 and no CRS data are available for this period. Moreover, the incompleteness of CRS data may hinder representativeness and, thereby, complicates the interpretability of excess deaths estimates relying on CRS data. The more nationally representative sample registration system (SRS) is often used to adjust for missing death information in CRS but SRS data are not yet available for 2020 and 2021. Lastly, while we use the latest available nationwide serosurvey to obtain an age-adjusted infections estimate in computing the IFR for SARS-CoV-2, we acknowledge that this approach does not incorporate factors of waning immunity and re-infections. We conjecture that, if such components were able to be accounted for, the denominator of the IFR (estimated infections) may have been larger and thereby the IFR will be attenuated to a degree.*”

R1.7 Specific Comments: What is the relationship between this paper and reference (1) Zimmermann et al? It would be helpful if the authors could clarify whether this paper is a presentation of the key findings of their earlier work, whether it includes further data and analysis, and so forth.

**Response:** We thank the reviewer for the question. In comparison to Zimmermann et al. (2021), the present analysis has more published data that in turn allows us to further qualitatively compare by disease transmission-based versus excess deaths studies, and more robustly meta-analyze excess deaths-based infection fatality rate estimates, with more data being available over two years since the start of the pandemic. We have added a sentence in the discussion at lines 137-140 that is related to this point as follows: “*Over two years since the start of the pandemic, numerous peer-reviewed studies have focused on understanding the actual death toll of SARS-CoV-2 in India, primarily either via excess deaths or disease transmission-focused modeling, enabling the meta-analysis herein of the 11 identified excess deaths estimates.*”

R1.8 Specific Comments: The line: "Supplementary Figure 2 shows a nationwide pooled IFR 2 estimate for India of 0.499%" does not seem to accord with Figure 2, where the pooled estimate appears to be 0.44%.

**Response:** We thank the reviewer for the comments. We have removed the former Supplementary Figure 2, as it is no longer relevant in the updated meta-analysis. To elaborate, the IFR estimates in Supplementary Figure 2 and Figure 2 of 0.499% and 0.440% differed because the former was calculated using the upper bound from included studies of available death underreporting factors, whereas the latter was calculated using the lower bound of death underreporting factors. However, as mentioned, we have removed the second forest plot that was Supplementary Figure 2 as we now directly incorporate the death uncertainty into the 95% confidence interval bounds for each included study in the meta-analysis (see updated Fig 2 above in our earlier response to R1.4, and further details on meta-analysis methodology in the supplementary materials Methods S2).

R1.9 Specific Comments: IFR1 (i.e., IFR based on recorded COVID-19 deaths) is fairly easy to calculate, but the origin of the IFR1 estimates quoted in the paper are not clear. If they are national estimates, then they are too high. For example, taking national level reported COVID-19 deaths, IFR1=0.1% would be consistent with an infection rate of a mere 34%, considerably lower than the estimates from recent serosurveys. The source of this mismatch requires clarification. Data from a few well-surveilled urban localities generally gives much higher values of IFR1 - is this the source of the high IFR1 estimate? If so, the aggregate IFR1 estimate should not be used to provide national estimates of URF (D). These matters are vitally important from the point of view of understanding the scale, geographical variation, and reasons behind COVID-19 death under-reporting in India.

**Response:** We thank the reviewer for the comments. The reviewer’s comments prompted us to clarify the following point in the manuscript. In particular, we have further focused the language in the manuscript on IFR_2_, although we keep IFR_1_ in the equations box in the methods section. This is because, while IFR_1_ was making the material more complex, some early studies present IFR_1_ (in other words present an infection fatality rate that uses only reported deaths as the numerator) and therefore, we have kept this term for discussion purposes.

R1.10 Specific Comments: Although the aggregate estimates of IFR2 are plausible, the calculations which underlie the individual estimates are not transparent. For example, some estimates are based on prevalence estimates in Murhekar et al (June 2020); but it is unclear what mortality data has been used as the numerator in these estimates. Do many of the papers include mortality data up to June-July 2020? If so, how reliable are these figures given, for example, the various delays and disruption to death registration during national lockdown? It would seem that a multiplicity of possible errors make the early estimates highly unreliable.

**Response:** We thank the reviewer for the comments. We similarly agree that death registration during the nationwide lockdown in the early phases of the pandemic may have resulted in less reliable estimates. In the updated meta-analysis results, we employ a time period criterion of included studies being through June 2021, in other words that studies must encompass the peak of the second wave (in May 2021) of COVID-19 within India. Lastly, we use the latest estimate that is reported from a given study. In turn, the meta-analyzed IFR estimates use the prevalence estimate of 67.6% from the latest 4^th^ nationwide serosurvey in 14 June-6 July 2021 (and prior to the roll-out of the vaccination program). Also, with additional available studies, we now do identify some peer-reviewed, published studies that directly provide a nationwide IFR_2_ for India, such as Barber et al (2022).

- Barber, R. M., Sorensen, R. J., Pigott, D. M., Bisignano, C., Carter, A., Amlag, J. O., ... & Murray, C. J. (2022). Estimating global, regional, and national daily and cumulative infections with SARS-CoV-2 through Nov 14, 2021: a statistical analysis. *The Lancet*.

R1.11 Specific Comments: In Table 1, the Wave 2 estimate for Banaji and Gupta is empty, but the preprint does include a point estimate (11.3) of the death underreporting factor during March-May 2021 in the subsection "Increasing under-ascertainment of COVID-19 deaths?" Is there a reason this was omitted?

**Response:** We thank the reviewer for this recommendation. We now have a uniform time period of studies through at least June 2021 and extending to December 2021, and no longer stratify by Wave 1 and Wave 2 in Table 1 and Fig 2. In other words, we instead focus on the latest overall estimates (as described in response to R1.10 above). Please see updated Table 1 in our response to R1.1 and updated Fig 2 in the response to R1.4 above.

R1.12 Specific Comments: In Figure 3, an IFR2 estimate for India of 0.135% is given based on IHME projections. But the IHME seemed to estimate 2.8M total deaths on Sept. 20, 2021, and a 66% infection rate, which would together give an IFR2 estimate of around 0.3%. Could the authors clarify the source of the IFR2 estimate of 0.135% attributed to the IHME?

**Response:** We thank the reviewer for the recommendations. As mentioned in a previous response, we have included an updated comparison across India, US, and Brazil that is in the body of the main-text in the discussion section, and we have removed Figure 3 from the report. We now compare excess death estimates for India, US, and Brazil, respectively, from Wang et al. (2022) and WHO, as well as we compare IFR estimates from Barber et al. (2022). For a direct quote of the updated in-text country comparison, see our response to R1.5 above.

- Barber, R. M., Sorensen, R. J., Pigott, D. M., Bisignano, C., Carter, A., Amlag, J. O., ... & Murray, C. J. (2022). Estimating global, regional, and national daily and cumulative infections with SARS-CoV-2 through Nov 14, 2021: a statistical analysis. *The Lancet*.
- Wang, H., Paulson, K. R., Pease, S. A., Watson, S., Comfort, H., Zheng, P., ... & Murray, C. J. (2022). Estimating excess mortality due to the COVID-19 pandemic: a systematic analysis of COVID-19-related mortality, 2020–21. *The Lancet*.
- Knutson, V., Aleshin-Guendel, S., Karlinsky, A., Msemburi, W., & Wakefield, J. (2022). Estimating Global and Country-Specific Excess Mortality During the COVID-19 Pandemic. arXiv preprint arXiv:2205.09081. (WHO reference)

**Reviewer #2 comments:**

The studies reviewed could be updated and expanded:

R2.1 Comments: The following study has India data which could be incorporated:

Wang H, Paulson KR, Pease SA, Watson S, Comfort H, Zheng P, Aravkin AY, Bisignano C, Barber RM, Alam T, Fuller JE. Estimating excess mortality due to the COVID-19 pandemic: a systematic analysis of COVID-19-related mortality, 2020–21. The Lancet. 2022 Mar 10.

**Response:** We thank the reviewer for this suggestion. We have included the Wang et al. (2022) estimates in the updated meta-analysis results. This change is reflected in the updated Table 1 and the updated Fig 2, which are included herein in above responses to R1.1 and R1.4, respectively.

R2.2 Comments: Your fourth reference by Deshmukh doesn’t list the title of the report:

Y. Deshmukh, W. Suraweera, C. Tumbe, A. Bhowmick, S. Sharma, P. Novosad, S.

H. Fu, L. Newcombe, H. Gelband, P. Brown, P. Jha, medRxiv, in press,

doi:10.1101/2021.07.20.21260872.

**Response:** We thank the reviewer for this suggestion. We now include the Jha et al. (2022) estimates in the updated meta-analysis results, in place of the previous version that is cited above (Deshmuk et al., 2021).

R2.3 Comments: I think that study is the same is this one by Jha, which is now published:

Jha P, Deshmukh Y, Tumbe C, Suraweera W, Bhowmick A, Sharma S, Novosad P, Fu SH, Newcombe L, Gelband H, Brown P. COVID mortality in India: National survey data and health facility deaths. Science. 2022 Jan 6:eabm5154.

**Response:** We thank the reviewer for the comments. As noted in the response to the above comment, we now include the Jha et al. (2022) estimate that is through November 2021 (i.e., their CVoter estimate). We do not include their CRS estimate through May 2021 because it does not meet our time period cut-off of through June 2021, to largely capture the second SARS-CoV-2 wave in India. We tried to make this reasoning clear by including a footnote on Table 1 to this effect, and by including asterisks in Table 1 to denote estimates that are included in the meta-analysis (and, thereby, are shown in the forest plot Fig 2). The updated Table 1 and the updated Fig 2 are included in above responses to R1.1 and R1.4, respectively.

R2.4 Comments: The study by Leffler et al is now published in a journal:

Preliminary Analysis of Excess Mortality in India During the COVID-19 Pandemic.

Leffler CT, Lykins V JD, Das S, Yang E, Konda S.

Am J Trop Med Hyg. 2022 Apr 4:tpmd210864. doi: 10.4269/ajtmh.21-0864. Online ahead of print. PMID: 35378508.

**Response:** We thank the reviewer for the comments. We now include the published estimates and citation for Leffler et al. (2022).

R2.5 Comments: The published version of the study by Leffler now has mortality data through August 31, 2021.

**Response:** We thank the reviewer for the comments. As noted in the comment above, we now include the published estimates for Leffler et al. (2022) through August 2021 in the updated meta-analysis. This change is reflected in the updated Table 1 and updated Fig 2, included in above responses to R1.1 and R1.4, respectively.

R2.6 Comments: The study by Guilmoto is now published:

Christophe Z Guilmoto. An alternative estimation of the death toll of the Covid-19 pandemic in India., PLoS One. 2022 Feb 16;17(2):e0263187. doi: 10.1371/journal.pone.0263187. eCollection 2022.

**Response:** We thank the reviewer for the comments. We now include in the updated meta-analysis the published estimates for Guilmoto et al. (2022) that the reviewer cited above.

**Reviewer #3 comments:**

The study appears an important meta-analysis of model and excess-deaths based studies of the pandemic's death toll in India, and using undercounting-ratios of infections based on seroprevalence studies, what a plausible range of the covid-19 IFR in India might be.

I believe that provided my below recommendations are followed and/or comments addressed, the study should be published and would be a valuable contribution. (This is also my understanding of the selection "minor revision"). I defer to discipline specialists on the fitness of article to journal. I also defer to discipline specialists on the statistical methodology employed, and the soundness and completeness of the replication package.

My comments will be restricted to the data used, and a few of the assumptions underlying the IFR estimations, and presentation. My apologies if I missed something obvious, including elements which address the concerns I raise below.

**Response:** We thank the reviewer for the positive comments.

R3.1 Comments: The Anand et al study has three estimates, but the study as far as I can tell only includes one of these. I would have liked to have seen an explicit justification of the choice of which to use. While I see a clear reason to exclude the IFR-based estimate, I wonder why the estimation based on the civil-state registration system was not included.

**Response:** We thank the reviewer for the suggestion. We now include all three excess deaths estimates for Anand et al (2021) that are from three distinct data sources and are through June 2021. This change is reflected in both the updated Table 1 and updated Fig 2, which are provided in above responses to R1.1 and R1.4, respectively.

R3.2 Comments: Acknowledging that I am an interested party, I find it puzzling that The Economist's estimates of excess deaths in India are not included, despite their global model being widely cited in other academic research (including that cited here), and having estimates on both the first and second wave.

**Response:** We thank the reviewer for the suggestion. We now include *The Economist* excess deaths estimates for India from December 31, 2021 in the meta-analysis. This change is reflected in the updated Table 1 and Fig 2, included in above responses to R1.1 and R1.4, respectively.

R3.3 Comments: Sero-prevalence based estimates are problematic beyond the early stages of the pandemic, because sero-reversion means that infections become progressively less probable to be detected, and because re-infections are possible. (They are also problematic in many contexts because of vaccines.) This should be acknowledged explicitly, and the implications discussed.

**Response:** We thank the reviewer for this important consideration. We have added a note within the limitations in the discussion section on this point at lines 162-169: “*Lastly, while we use the latest available nationwide serosurvey to obtain an age-adjusted infections estimate in computing the IFR for SARS-CoV-2, we acknowledge that this approach does not incorporate factors of waning immunity and re-infections. If such components were able to be accounted for, the denominator of the IFR (estimated infections) may have been larger and thereby the true IFR will be attenuated to a degree. Such limitations inherent to sero-surveillance studies also include sero-reversion which concerns reduced detection of SARS-CoV-2 antibodies and leads to an upward bias in IFR estimates (8).*”

R3.4 Comments: I found it hard to follow where infection estimates were from - I would have liked something like what was done for table 1 except for infection estimates. Table 2 was confusing to me - I would try to improve this presentation.

**Response:** We thank the reviewer for their recommendation. We have included a statement at the top of Table 1 that states the source for the infection estimates, which were previously listed in the left-hand column of Fig 2 (please see updated Table 1 above in response to R1.1). We have also included a flow diagram that demonstrates how we use infections and mortality data extracted from the included studies to obtain IFR estimates, that we meta-analyze (see in the supplementary materials Fig S1 included above also in our response to R1.1).

R3.5 Comments: The use of the IHME estimate of infections is problematic in figure 3, because, unless I am mistaken, this estimate is based on an estimated IFR and estimated death rates, meaning the implied IFR is "baked in". The figure is also unclear (that is, if the bars are meant to mutually exclusive).

**Response:** As we mention in responses to Reviewer 1 above, we have removed the Figure 3 and instead we have included the comparison of infection fatality rates from India, US, and Brazil in the text body of the discussion section. We have included the related excerpt from the manuscript above in response to R1.5. Moreover, we now rely on estimates for India, US, and Brazil, respectively, from Barber et al. (2022), Wang et al. (2022) and WHO.

- Barber, R. M., Sorensen, R. J., Pigott, D. M., Bisignano, C., Carter, A., Amlag, J. O., ... & Murray, C. J. (2022). Estimating global, regional, and national daily and cumulative infections with SARS-CoV-2 through Nov 14, 2021: a statistical analysis. *The Lancet*.
- Wang, H., Paulson, K. R., Pease, S. A., Watson, S., Comfort, H., Zheng, P., ... & Murray, C. J. (2022). Estimating excess mortality due to the COVID-19 pandemic: a systematic analysis of COVID-19-related mortality, 2020–21. *The Lancet*.
- Knutson, V., Aleshin-Guendel, S., Karlinsky, A., Msemburi, W., & Wakefield, J. (2022). Estimating Global and Country-Specific Excess Mortality During the COVID-19 Pandemic. arXiv preprint arXiv:2205.09081.

R3.6 Comments: For reasons discussed elsewhere, see e.g. Andrew Gelman (2021), I would consider avoiding reliance on IHME estimates regarding the pandemic in general.

**Response:** We thank the reviewer for the comments. As we note in our responses to Reviewer 1, we agree that the IHME estimates being model-based and subject to other assumptions are outliers. Although we previously cautioned that the IHME estimates are considerably conservative in the discussion section, we concur and have removed Figure 3. As we note above, we currently use the published estimates from the following three global studies: Barber et al. (2022), Wang et al. (2022) and WHO, instead of the previously used IHME projections.

R3.7 Comments: My recommendation would be to drop this comparison to other the US and Brazil, and rather here summarize other estimates of IFR (of which there are many - see e.g. Brazeau et al 2020 and the papers citing this study).

**Response:** We thank the reviewer for their recommendation. We agree that an in-text discussion summarizing a comparison of other IFR estimates across countries is more appropriate. As mentioned above, we have removed Figure 3 and instead include a paragraph in the discussion section relying on the Barber et al. (2022), Wang et al. (2022) and WHO estimates. In light of the reviewer recommendation to include other IFR estimates, we compare the pooled IFR estimate against the Barber et al. (2022) estimate for India in the discussion section at lines 147-150: *“The pooled IFR2 estimate from COVID-specific transmission model-based studies is largely congruent to the estimated IFR of 0.3% (as of 14 November 2021) for India reported in the global IFR study by Barber et al. (2022) (7).*”

- Barber, R. M., Sorensen, R. J., Pigott, D. M., Bisignano, C., Carter, A., Amlag, J. O., ... & Murray, C. J. (2022). Estimating global, regional, and national daily and cumulative infections with SARS-CoV-2 through Nov 14, 2021: a statistical analysis. *The Lancet*.
- Wang, H., Paulson, K. R., Pease, S. A., Watson, S., Comfort, H., Zheng, P., ... & Murray, C. J. (2022). Estimating excess mortality due to the COVID-19 pandemic: a systematic analysis of COVID-19-related mortality, 2020–21. *The Lancet*.
- Knutson, V., Aleshin-Guendel, S., Karlinsky, A., Msemburi, W., & Wakefield, J. (2022). Estimating Global and Country-Specific Excess Mortality During the COVID-19 Pandemic. arXiv preprint arXiv:2205.09081.

R3.8 Comments: With regards to discussion on age structure, the following might be of interest: https://github.com/TheEconomist/covid-19-age-adjusted-ifr -- these also figure in The Economist's global model.

**Response:** We thank the reviewer for the suggestions. We have added a few sentences on the provided age-adjusted IFR source in the discussion section, where we note the importance of accounting for differences in age structures as it pertains to mortality estimation. This insertion is in the discussion section at lines 193-196: “*Recent studies underscore the importance of adjusting for age structures, when performing related deaths estimations. For example, The Economist recently made available an age-adjusted IFR source (github.com/TheEconomist/covid-19-age-adjusted-ifr), which is further incorporated into their published estimates (11).*”

R3.9 Comments: I find it strange that there is no mention of changes in IFR over time. This has been known to respond to health care capacity, treatment quality changes, vaccines, and variants. This must at a minimum be acknowledged.

**Response:** We thank the reviewer for this important consideration. We agree that the lethality of the virus is subject to multiple time-varying components, as is listed, and, in particular, the roll-out of vaccines in India starting in Jan 2021 and the changing variant landscape. We have included sentences in the discussion on these critical points at lines 198-203: “*Many of the included studies in this meta-analysis also sought to account for changes in deaths and subsequently changes in IFR over time often by incorporating as granular, longitudinal data as possible. This is important as the lethality of the virus is subject to multiple time-varying components, especially the roll-out of vaccines (starting in January 2021 within India), as well as the changing variant landscape wherein the milder SARS-CoV-2 variant Omicron and sub-lineages became dominant.*”

R3.10 Recommendations: Add justification of selection of which Anand et al study estimate to use

**Response:** We thank the reviewer for the comments. As mentioned in response to Reviewer 2, we now include all three excess deaths estimates for Anand et al (2021), that rely on three independent data sources and are through June 2021. All three Anand et al (2021) estimates are included in the meta-analysis and are now reflected in the updated Table 1 and Fig 2, included in above responses to R1.1 and R1.4, respectively.

R3.11 Recommendations: Clearer presentation of sources for infection estimates, and a discussion of their limitations (sero-reversion being an important one)

**Response:** We thank the reviewer for the suggestions. As we note above, we have changed the time period of coverage and now implement a cut-off of studies being at least through June 2021 and extending to December 2021. Likewise, we now only use the 4^th^ serosurvey in hopes of simplifying presentation. We have tried to clarify this point through including a statement in Table 1 on the source for the infection estimate (the 4^th^ nationwide serosurvey in India) and a data flow diagram (see in the supplementary materials Fig S1 included above in response to R1.1).

With respect to sero-reversion (as mentioned above), we have also included a sentence in the limitations within the discussion section concerning shortcomings of serosurveys in lines 167-169: “*Such limitations inherent to sero-surveillance studies also include sero-reversion which concerns reduced detection of SARS-CoV-2 antibodies and leads to an upward bias in IFR estimates (8).*”

R3.12 Recommendations: Include The Economist's estimate for India's death toll

**Response:** We thank the reviewer for the recommendations. As noted above, the updated meta-analysis results now include The Economist deaths estimate for India. Please see updated Table 1 and Fig 2 that are included in above responses to R1.1 and R1.4, respectively.

R3.13 Recommendations: Drop figure 3, which is both confusing, is based on a different approach than the rest of the study, and presents information which is already widely known

**Response:** We thank the reviewer for the recommendations. As noted above, we have removed Figure 3 and instead included an in-text comparison of countries to the discussion section that uses Barber et al. (2022), Wang et al. (2022) and WHO estimates. For the related excerpt from the manuscript, please see response to R1.5 above.

- Barber, R. M., Sorensen, R. J., Pigott, D. M., Bisignano, C., Carter, A., Amlag, J. O., ... & Murray, C. J. (2022). Estimating global, regional, and national daily and cumulative infections with SARS-CoV-2 through Nov 14, 2021: a statistical analysis. *The Lancet*.
- Wang, H., Paulson, K. R., Pease, S. A., Watson, S., Comfort, H., Zheng, P., ... & Murray, C. J. (2022). Estimating excess mortality due to the COVID-19 pandemic: a systematic analysis of COVID-19-related mortality, 2020–21. *The Lancet*.
- Knutson, V., Aleshin-Guendel, S., Karlinsky, A., Msemburi, W., & Wakefield, J. (2022). Estimating Global and Country-Specific Excess Mortality During the COVID-19 Pandemic. arXiv preprint arXiv:2205.09081.

R3.14 Recommendations: Replace this with a discussion, and perhaps a table, of how this compares to other studies' estimates of IFRs.

**Response:** We thank the reviewer for the recommendations. Please see the response above. Also, we now compare the pooled IFR estimate against the Barber et al. (2022) estimate for India in the discussion section at lines 147-150: *“The pooled IFR_2_ estimate from COVID-specific transmission model-based studies is largely congruent to the estimated IFR of 0.3% (as of 14 November 2021) for India reported in the global IFR study by Barber et al. (2022) (7).*”

R3.15 Recommendations: Consider including a mention of age-adjusted implied IFR by country.

**Response:** We thank the reviewer for the recommendations. As we note above in response to R3.8, we have included the following sentences citing *The Economist* age-adjusted IFR estimates in the discussion section at lines 193-196: “*Recent studies underscore the importance of adjusting for age structures, when performing related deaths estimations. For example, The Economist recently made available an age-adjusted IFR source (github.com/TheEconomist/covid-19-age-adjusted-ifr), which is further incorporated into their published estimates (11).*”

R3.16 Recommendations: Add to discussion the limitations of the study as it relates to varying IFRs over time: this should mention vaccines, the sars-cov-2 variants, treatment quality, and how this study is based on data from a certain such context, and how this context has changed.

Best of luck in revisions of this important research.

**Response:** We thank the reviewer for the recommendations. As we note above in response to R3.9, we have included a few sentences in the discussion section on the very important points the reviewer has raised with respect to changes in IFR over time. These sentences are included in the discussion section at lines 198-203: “*Many of the included studies in this meta-analysis also sought to account for changes in deaths and subsequently changes in IFR over time often by incorporating as granular, longitudinal data as possible. This is important as the lethality of the virus is subject to multiple time-varying components, especially the roll-out of vaccines (starting in January 2021 within India), as well as the changing variant landscape wherein the milder SARS-CoV-2 variant Omicron and sub-lineages became dominant.*”

**Reviewer #4 comments:**

R4.1 Comments: I wasn't totally sure of the methodology, and I see that estimates of the IFR can be obtained using lower bounds on under-reporting. But with a sampling-based approach (from the asymptotic distribution of your estimators), couldn't you obtain estimates of IFR that account for uncertainty in the underreporting?

**Response:** We thank the reviewer for the comments. In effort to clarify the IFR derivation, we have included a data flow diagram in the supplementary materials Fig S1 that shows how data from included studies is used to arrive at IFR estimates that are subsequently meta-analyzed. This new visualization is included above in our response to R1.1.

R4.2 Comments: It wasn't clear to me what was model-based and what wasn't. IHME and The Economist excess mortality estimates for India would be model-based, right?

**Response:** We thank the reviewer for the comments. We now include the published versions of Barber et al (2022) and Wang et al (2022) in place of the previous IHME estimates in Table 1. We now classify studies into three categories: excess deaths studies, disease transmission-based studies estimating unreported deaths, and disease transmission-based studies using reported deaths only. With regard to this grouping, as examples, *The Economist* and Wang et al (2022) estimates reflect all-cause excess deaths estimation and are categorized as excess deaths studies, for this reason. Whereas the Barber et al (2022) estimate reflects COVID-19 attributed deaths and is not considered all-cause. Moreover, for example, the Shewade et al (2021) and Zimmermann et al (2021) reflect COVID-19 related deaths estimates and estimate unreported deaths that are not reliant on excess deaths computations and, therefore, we classify such studies as disease transmission-based studies that account for unreported deaths.

R4.3 Abstract: ``Highly conservative'' could be misinterpreted as for the ratio (the IFR) but you are referring to the underreporting of numerator and denominator, which does not mean the ratio is highly conservative. To be clear, I'm not suggesting you report different IFRs, but am commenting on the wording.

**Response:** We thank the reviewer for the comments. We have clarified this point in the abstract by rewording to this effect at lines 53-55: “*Conservatively, by considering the lower bounds of these available estimates, we infer that approximately 95% of infections and 71% of deaths were not accounted for in the reported figures in India.*”

R4.4 Page 3: ``top three countries", via what metrics?

**Response:** We thank the reviewer for the recommendations. In the discussion section, we state that the three countries of India, US and Brazil are compared by the following metrics reported cumulative cases and reported cumulative deaths as of December 31, 2021. We include the following related comments within the discussion section in lines 172-176: “*Considering the three countries with the highest cumulative reported deaths (as of December 31, 2021), namely, India, Brazil, and the United States (in ascending order), the IFR2 (as of 14 November 2021) reported by Barber et al. (2022) appears to be the lowest in India (IFR2 of 0.3%) compared to the US (IFR2 of 0.9%) and Brazil (IFR2 of 0.5%) (7).*”

We also acknowledge the following about using the metric of cumulative deaths for the purpose of illustration in the discussion section at lines 185-188: “*While metrics are useful for evaluating public health policies, we caution against such crude comparisons based on a single metric. Although we use cumulative excess deaths as a measure of comparison in mortality ranking, population counts are not factored in and deaths per million may be preferrable in another context.*”

R4.5 Page 3: ``obscur". Typo.

**Response:** We thank the reviewer for the recommendations. We have changed the spelling accordingly to be “*obscure*”.

R4.6 Page 3: Give 3 decimal places for 0.44\% figure.

**Response:** We thank the reviewer for the recommendations. We now include only two decimal places in the IFR estimates throughout the in-text estimates, tables and figures provided in the updated meta-analysis, as this was the precision achievable given the included studies.

R4.7 Page 4: Is there a reason why for URF(C) you have "Total Cumulative Infections" in the numerator and "Cumulative Cases" in the denominator. Is it important to distinguish between ``infections" and ``cases"? And no ``Total" in the denominator.

**Response:** We thank the reviewer for the important recommendations. We have defined the terms Total Cumulative Infections and Cumulative Cases in the supplementary materials Methods S2. We confirm that the understanding of the reviewer is correct with respect to the importance to distinguish between infections and cases. Briefly, the denominator for URF (C) is “Reported Cumulative Cases” which we distinguish from “Estimated Total Cumulative Infections” as the term *Total* is intended to reinforce the distinction between infections and cases, with the former being the true estimated number of COVID-19 infections and the latter being the reported number of COVID-19 cases. In other words, existent studies have sought to estimate the former often, rigorously via household-based sero-surveillance studies (e.g., India’s nationwide serosurvey, in the case of a countrywide effort). As included in R1.1 above, we have included in the supplementary materials Fig S1 in hopes of further clarifying this important point raised by the reviewer.

R4.8 Table 1: I didn't fine this table very easy to take in. Having a column labeled ``excess deaths" would help.

**Response:** We thank the reviewer for the recommendations. We have changed the labeling in Table 1 to reflect excess deaths by categorizing the meta-analyzed studies as excess deaths studies and the non-meta-analyzed studies as disease transmission-based studies. We have included the updated Table 1 in response to R1.1 above.
